# Pueperal Mania

**Published:** 1874-02

**Authors:** Thomas H. Mayo

**Affiliations:** Mississippi


### PUEPERAL MANIA.

BY THOS. H. MAYO, M.D., OF MISSISSIPPI.

*Read before the Columbus and Lowndes County Medical Society, Mississippi.*

This disease is so nearly allied to insanity, that judging by the mental symptoms alone, we would be unable to distinguish it, being liberal to the same varieties as insanity. It usually commences from the third to the thirtieth day after confinement, although it may occur early in pregnancy, also in the latter months of lactation, produced as is supposed, from the exhaustive effect of continuing that function too long.

The pathology of the disease is not well settled, the cadavour failing to give any pathological changes, such as would satisfactorily account for the symptoms of those we find most predisposed to this affection, are the nervous, excitable temperament—it does not seem to be governed by any accidents which occur in labor—it as often follows easy, natural labor as the difficult and exhausting.

The earliest observable symptoms is restlessness, with impatience and great disposition to find fault with those around her—is perhaps less suspicous than in cases of insanity, this is followed by complete insomnia for one or two nights before there is any noticeable aberation of mind.

Those who are attacked soon after confinement have usually pleasurable excitement, with great disposition to incoherent and incessant talking, frequently repeating a word or sentence for several minutes or until some other sound is heard, which she at once takes up—this is continued for days without intermission—the movement of her limbs seem incontrolable and irregular, pulse frequent and full, but soft, skin bathed in perspiration though hot, bowels constipated, stomach is not irritable, and this indicates the distinction between this disease and cerebral inflamation.. There is no under heat of the head, nor does the patient complain of headache, appetite is various, though they will usually take such food and medicine as is presented to them, these symptoms may last for a few days, and may continue for several weeks.

Should the disease occcur after the first month, it is more liable to assume the form of melancholia. The patient is gloomy, apprehends some inpending evil to herself and family, is suspicious of her best friends and nearest relations, there is more prostration, less frequency of the pulse and heat of the skin, insomnia is not so complete, nor is there such an incessant restlessness, in fact the patient will frequently sit for hours without speaking or moving, and when they speak it is only in a subdued whisper, as though afraid of being overheard.

In neither case will such patient notice the child, but seem oblivious to its existence.

The prognosis in puerperal mania is generally favorable, but when the pulse is very freqent and the patient shows much weakness, there is danger of sinking from exhaustion, but when the attack does not assume this form, it is much more frequently shortened either by self limitation or treatment, than melancholia, most usually terminating in a few weeks or months, while in melancholia the case may continue for a year or more, and rarely proving fatal, and in some cases results in permanent insanity.

Whether such instances were the result of purperal mania or insanity, from the inception, we think may be fairly questionable, from the fact before stated, that there are no pathognomonic symptoms by which the two disorders can be distinguished.

During the continuance of this disease there are no prominent uterine symptoms which will enable us to account for the wonderful nervous disturbance. The lochia is sometimes suppressed or lessened, the secretion of milk is generally suppresed or does not appear.

Some authors are disposed to attribute the disease to severe nervous excitability before monopolized by the uterus, which should now be concentrated on the mammary organs, but instead of this, it involves the brain, but this theory would leave out all those cases which occur before parturition, as well as those occurring during lactation. At present, we are as ignorant of the pathology of this form of disease as we are of insanity.

In the treatment of puerperal mania, we have three indications to fulfill, viz: Correct the secretions, procure sleep and sustain the patient.

The first can probably be secured by a purgative dose of calomel, followed if necessary with oil and turpentine; the mercury should be repeated in a few days, should the tongue not clean off, the bowels should be moved moderately, at least every other day; active purgation must be avoided, though usually it requires the free use of active cathartics to move the bowels at all, the system being particularly insensible to medicines. In the mean time we should endeavor to quiet nervous excitement and procure sleep—this is the great curative agent—all other indications are subsidiary or preparatory to this. If you can by any means secure eight to twelve hours uninterrupted sleep, you will in nearly every case find the intellect clearer and the patient more calm. The bromide potassa, in half drachm (ʒss.) doses, repeated every four hours will tend to quiet nervousness and prepare the system for the more powerful hypnotics, of these, the most reliable as well as saftest, is opium, in free doses. We prefer the acetate it is are less apt to produce primary excitement followed some hours after by nausea.

In puerperal mania we find many cases that do not bear opiates well; one of the best substitutes is chloral hydrate in half drachm (ʒss.) doses repeated every half hour or hour, until two drachms are taken—the patient should be carefully watched while under full doses of chloral hydrate, as there is much danger of producing paralysis of the pneumogastric nerve, followed by cessation of respiration and stopping the heart's action resulting fatally.

We usually combine acetate morphia with the chloral; the combination acts more satisfactorily and is more permanent. Other nervous sedatives are too weak to be relied upon. As soon as the tongue is clean and bowels are in a soluble condition, we may use tonics, as iron with bitters or comp. tr. barks.

Do not use quinine unless indicated by some marked periodicity, as it tends to increase nervous disturbance. Have seen valerianate quinine used in small doses, but with doubtful success.

In the early stages of the disease when the pulse is very frequent, the tr. gelseminum or tr. veratrum viridi in sufficient doses to moderate the hearts action would perhaps be beneficial. Should the secretion from the kidneys fail, we must substitute digitalis—during the treatment it is necessary to examine frequently the condition of the bladder, to prevent distension, even while the nurses may assure you that the patient has passed urine in abundance. In this class of patients, it is essential to use the catheter daily, and should your patient become sufficiently quiet and manageable to take it, let her have an abundance of mild nutritious food, and direct your efforts to establish the secretion, or if too late for that, then the menstural discharge.

